# Profiling Maternal Behavior Responses During Whole-Brain Imaging

**DOI:** 10.3791/67112

**Published:** 2025-01-24

**Authors:** Briana R. McRae, Amir Lawen, Itamar Kahn, Bianca J. Marlin

**Affiliations:** 1 Mortimer B. Zuckerman Mind Brain Behavior Institute, Columbia University; 2 Department of Neuroscience, Columbia University; 3 Department of Psychology, Columbia University; 4 Howard Hughes Medical Institute, Columbia University

## Abstract

Recent advancements in whole-brain imaging tools have enabled neuroscientists to investigate how coordinated brain activity processes external cues, influencing internal state changes and eliciting behavioral responses. For example, functional magnetic resonance imaging (fMRI) is a noninvasive technique that allows for the measurement of whole-brain activity in awake, behaving mice using the blood oxygenation-level-dependent (BOLD) response. However, to fully understand BOLD responses evoked by external stimuli, it is crucial that experimenters also assess behavioral responses during scans. The MRI environment poses challenges to this goal, rendering commonly employed methods of behavioral monitoring incompatible. These challenges include (1) a restricted field of view and (2) the limited availability of equipment without ferromagnetic components. Presented here is a behavioral video analysis pipeline that overcomes these limitations by extracting valuable information from videos acquired within these environmental constraints, enabling the evaluation of behavior during the acquisition of whole-brain neural data. Employing methods such as optical flow estimation and dimensionality reduction, robust differences can be detected in behavioral responses to stimuli presented during fMRI scans. For example, representative results suggest that mouse pup vocalizations, but not pure tones, evoke significantly different behavioral responses in maternal versus virgin female mice. Moving forward, this behavioral analysis pipeline, initially tailored to overcome challenges in fMRI experiments, can be extended to various neural recording methods, providing versatile behavioral monitoring in constrained environments. The coordinated evaluation of behavioral and neural responses will offer a more comprehensive understanding of how the perception of stimuli leads to the coordination of complex behavioral outputs.

## Introduction

Monitoring behavioral responses during neural recordings is essential for understanding stimulus-evoked coordinated activity across the brain. In the event that animals are not predicted to respond to stimuli in a specific and goal-oriented manner, observing uninstructed behaviors can offer insights into how external cues inform their internal states^1,2^. Recent advancements in neuroimaging tools, such as wide-field calcium imaging and functional magnetic resonance imaging (fMRI), have enabled neuroscientists to expand investigations beyond singular brain regions. However, to achieve a more comprehensive understanding of such high-dimensional neural data, the ability to evaluate the behavioral outputs of these complex patterns must also advance accordingly.

State-of-the-art methods for characterizing task-instructed behaviors are widely used in neuroscience research, including temperature and pressure sensors to detect sniffs^3,4^, light beams to detect licks^5^, and markerless pose estimation to track predetermined body parts^6^. However, the data-driven evaluation of uninstructed behavioral patterns remains a challenge in the field^7^. While methods for data-driven behavioral analysis are advancing rapidly, existing methods typically require substantial computational power, specialized equipment, or a particularly clear view of the animal^2,6,8,9^. Presented here is a behavioral video analysis pipeline that is easily amenable to any videography data and allows for the extraction of valuable behavioral measures from videos acquired during passive stimulation.

This behavioral analysis pipeline was designed to be compatible with the coordinated acquisition of neural and video data in head-fixed animals exposed to a broad range of external stimuli and multiple recording environments, even those with considerable constraints. For example, the MRI environment poses specific challenges to behavioral monitoring, including a restricted field of view and limited availability of equipment without ferromagnetic components. These constraints render commonly employed methods incompatible, leaving measures such as head motion (i.e., how the brain moves between volume acquisitions) to be among the most accessible yet limited ways to assess bodily responses during scans^10^. By overcoming these limitations, this protocol facilitated the analysis of MR-compatible videography data, showing that female mice with different maternal experiences exhibit distinct behavioral responses to auditory stimuli. Mothers and virgins were presented with auditory stimuli during fMRI scans, including distressed pup vocalizations (“pup calls”) and pure tones. These stimuli were presented passively, thus without any instructed behavioral outputs. While mothers were predicted to show heightened responses to pup calls compared to virgins, there is little literature on how female mice respond to infant cues in a head-fixed environment. Therefore, there was no specific behavioral output to track *a priori*, making this experiment the perfect test for the proposed data-driven behavioral analyses. Employing methods such as optical flow estimation and dimensionality reduction, group differences in behavioral responses were detected, both in magnitude and spatial manifestation.

Moving forward, the coordinated evaluation of behavioral and neural responses can be leveraged to achieve a more comprehensive understanding of how external stimuli alter internal states and inform complex behavioral outputs. This understanding should extend to stimuli presented in the absence of imposed tasks and instructed behaviors. The analysis pipeline presented here can be extended to various neural recording techniques, providing versatile behavioral monitoring even in constrained environments.

### Protocol

All animal experiments were performed according to the protocols approved by the Columbia University Institutional Animal Care and Use Committee (IACUC), and all methods were carried out in accordance with relevant guidelines, regulations, and recommendations. The details of the equipment and software used are listed in the Table of Materials.

#### 1. Software

1. Download **MATLAB** from the Mathworks website.
  NOTE: The code that comprises this analysis pipeline is executable solely through MATLAB and was specifically written to be compatible with MATLAB 2023a.
2. This pipeline requires the Computer Vision Toolbox and the Image Processing Toolbox within MATLAB. Add the toolboxes to MATLAB add-ons by clicking on **Manage Add-Ons, Get Add-Ons**, and then search for and add them.
  NOTE: Five MATLAB scripts are provided as supplementary files, titled as follows: script1_videocoreg.m (Supplementary Coding File 1), script2_optflow_roiselect.m (Supplementary Coding File 2), script3_optflow_analysis.m (Supplementary Coding File 3), script4_optflow_pca.m (Supplementary Coding File 4), and script5_optflow_pca_analysis.m (Supplementary Coding File 5). Two example videos (Video 1 and Video 2) and their corresponding event timing files (Supplementary File 1 and Supplementary File 2) are also provided. It is recommended to first run through the pipeline with the example data.
  CAUTION: Beware of any comments in the code that include an exclamation mark, as these are areas that require action before execution. For example, there are comments such as “edit the code block below!” to highlight where edits must be made to fit file structures, experiment details, or analysis needs. These choices are set by default to fit the two provided example videos but will need adjustments once one starts to run the pipeline with their own data.
3. To run each section of each script, click on **Run and Advance** on the **Editor** menu.

#### 2. Video coregistration

NOTE: The first provided script in this analysis pipeline is script1_videocoreg.m (Supplementary Coding File 1), which spatially aligns all videos to each other *via* coregistration. The inputs for this script are the raw videos, and the main outputs are the transformed videos.

**Figure 1:**
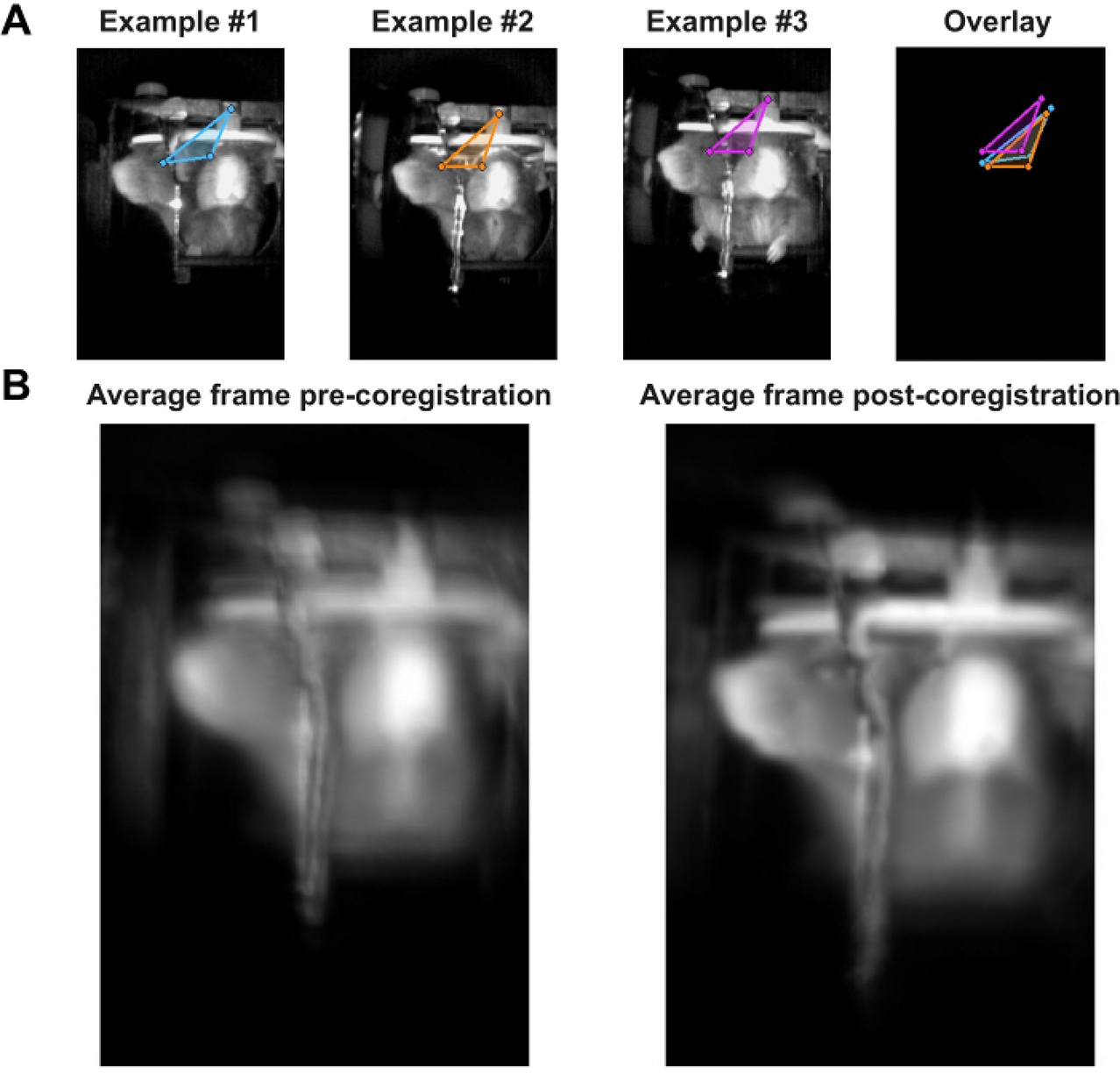
Video coregistration. (**A**) Examples of video coregistration depicting predetermined features (center of the head-post, frontal view of the right eye, profile view of the right eye) labeled in three frames, each taken from different videos of different animals. Note in the overlay how the three points were not aligned, demonstrating how the behavioral set-up changed slightly between data acquisition sessions. (**B**) The average frame across the last 10 s of each video before (left) and after (right) performing video coregistration.

1. Edit Sections 1, 2, 3, 4, 5, and 6 of the script where indicated to fit data structures.
2. Run Section 1.
3. Choose three points in each video to label, as in Figure 1A. These three points will be used to coregister all frames to each other. Run Section 2 of the script to pull up the first frame of each video and use the mouse to click once on the **first two points**, then double-click on the **third point**. After the last video’s points have been selected, press **Enter**.
4. Run Section 3 and Section 4.
5. Set the flag **aligned** to **0** and run Section 5. Then, set the flag **aligned** to **1** and run Section 5 again.
6. Run Section 6 and compare the resulting side-by-side figure to Figure 1B. Any aberrations observed in the non-aligned average image on the left should be lessened in the aligned average image on the right.

#### 3. Optical flow estimation: Full FOV

**Figure 2:**
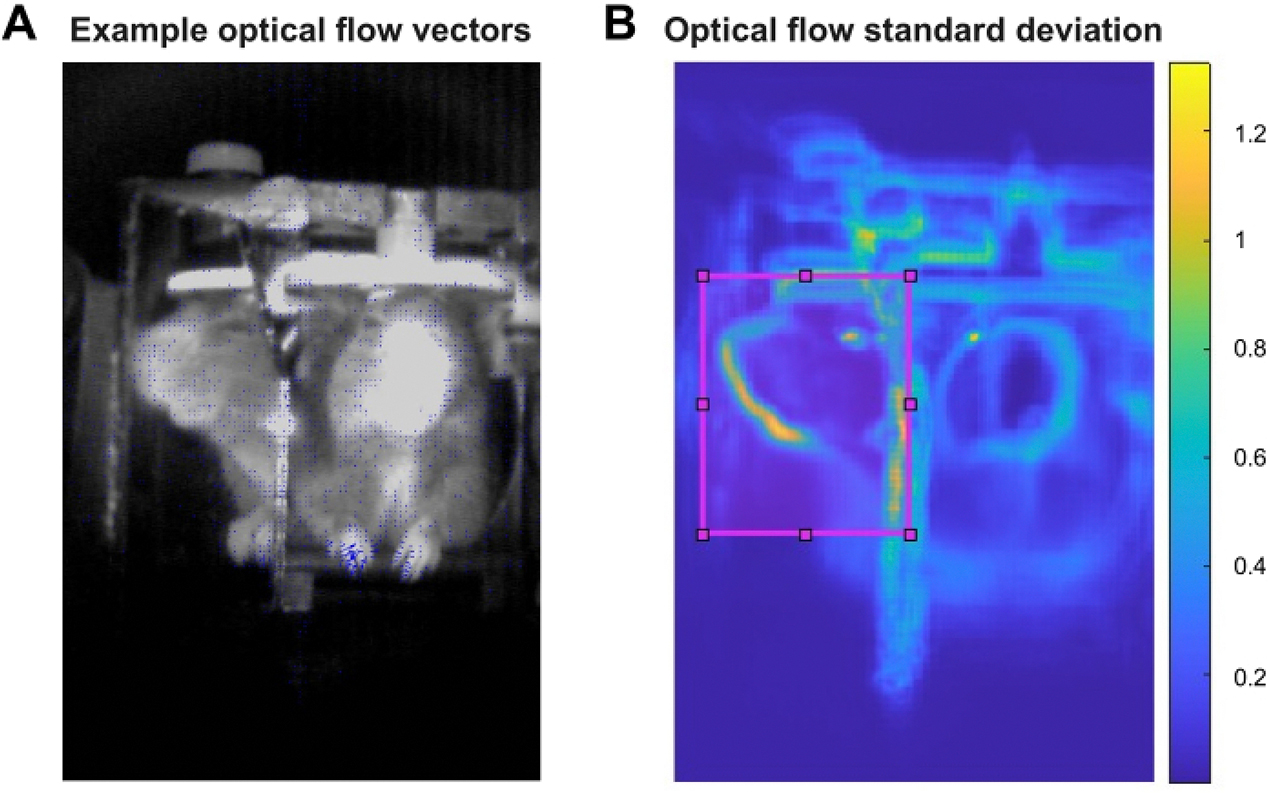
Optical flow estimation. (**A**) Example frame with relative optical flow vectors overlaid in blue. (**B**) The average pixel-wise standard deviation of optical flow across all videos. The chosen ROI around the profile view of the animal’s face *via* the mirror is outlined in magenta. The color bar corresponds to the standard deviation.

NOTE: In order to assess movement through the scan videos, optical flow needs to be estimated for each transformed video. This can be done for the full field-of-view (FOV) of each video with the provided script script2_optflow_roiselect.m (Supplementary Coding File 2). The inputs for this script are the transformed videos, and the outputs are 3D matrices indicating the optical flow magnitude of each pixel across time for each video. Figure 2A shows an example frame with optical flow vectors overlaid in blue, where each vector’s length represents the relative optical flow magnitude of that pixel. Saving the outputs as videos was found to be more efficient than saving them as 3D matrices.

1. Edit Section 1 of the script where indicated to fit data structures.
2. Run Section 1.

#### 4. ROI selection

NOTE: The following analysis steps are made much more manageable in terms of computational load and data storage needs by selecting a region of interest (ROI) within the full FOV on which to focus the analysis. The ROI can be selected according to the experiment and behaviors of interest or through a more data-driven approach. For the representative results presented here, the standard deviation of the optical flow magnitude of each pixel in the full FOV was calculated with the script script2_optflow_roiselect.m, Section 2 (Supplementary Coding File 2). The inputs for this part of the script are the outputs of Section 1, and the main output is a visualization of the standard deviation of the optical flow magnitude across all pixels across all videos. In the representative results presented, the mirror FOV showed a relatively high standard deviation of optical flow, which guided the demonstrated choice of ROI.

1. Edit Section 2 of the script where indicated to fit data structures.
2. Run Section 2 and use the resulting image to see where optical flow fluctuates most dramatically in the analyzed videos. An example of this image is shown in Figure 2B.

#### 5. Optical flow quantification: ROI

NOTE: Section 3 of the script script2_optflow_roiselect.m (Supplementary Coding File 2) allows the user to select an ROI with a drawing tool and save the boundary coordinates. The inputs for this part of the script are the outputs of Section 1, and the outputs are 1D vectors indicating the average optical flow magnitude of the ROI over time for each video.

1. Edit Section 3 of the script where indicated to fit data structures and analysis needs.
  NOTE: There are a few options regarding how to proceed with ROI selection. Choose one option:
  1. Option 1: Analyze the full video FOV.
    1. Set the flag **selectROI** to **0** and set **provideROI** to **0**.
  2. Option 2: Analyze a new ROI.
    1. Set the flag **selectROI** to **1** and set **provideROI** to **0**. Then edit **newCoordsName**.
  3. Option 3: Analyze a previously drawn ROI.
    NOTE: Only select this option if one has previously run this code and created an ROI.
    1. Set the flag **selectROI** to **0** and set **provideROI** to **1**. Then, edit **inputCoords** to provide a predetermined set of coordinates.
2. Run Section 3.

#### 6. Optical flow magnitude comparison

NOTE: The provided script script3_optflow_analysis.m (Supplementary Coding File 3) requires the most specialization to fit the user’s data. The main inputs are the 1D vectors indicating the average optical flow magnitude of the ROI over time for each video, and when combined appropriately with event onsets and group/condition information, the main outputs are statistical comparisons that can be tailored to analysis interests.

**Figure 3:**
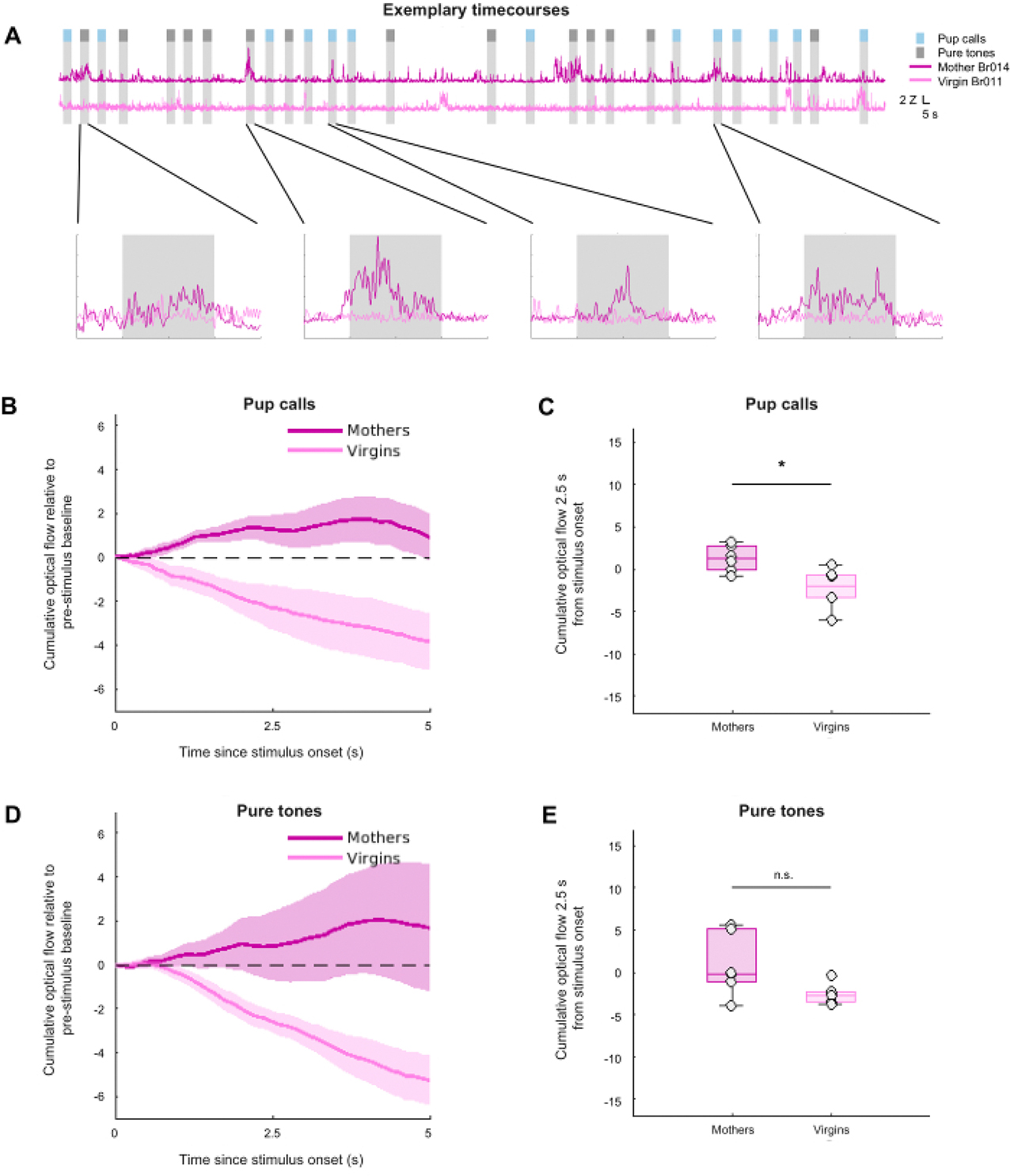
Visualization and comparison of optical flow across groups and conditions. (**A**) Exemplary Z-scored and 5 Hz lowpass filtered time series of optical flow for one video of a mother and one video of a virgin. (**B**) and (**D**) Cumulative optical flow measured during the stimulus period of pup calls (**B**) and pure tones (**D**). Shading represents the standard error of the mean (SEM). (**C**) and (**E**) Cumulative optical flow during the first 2.5 s of stimulus presentation for pup calls (**C**) and pure tones (**E**). * Indicates *p* < 0.05, between-group Mann-Whitney U test (pup calls: *p* = 0.026; pure tones: *p* = 0.093), N = 6 per group.

1. Edit Sections 1–3 of the script to fit data structures and analysis needs.
2. Set the analysis options flags **zsc, blrm, blzsc, demeanPerTrial**, and **applyLPfilter** to **0** or **1** as desired in Section 1. In the representative results presented, **zsc, blrm,** and **applyLPfilter** were set to **1**, and all other options were set to **0**.
3. Set **LPfilter** to a number representing the desired lowpass filter in hertz (Hz). In the representative results presented, a 5 Hz lowpass filter was applied, as behavioral responses were not expected to fluctuate at a rate faster than 5 Hz.
4. Run Sections 1–3. Section 3 should produce plots for group average optical flow time series, group average cumulative optical flow, and summary cumulative optical flow, including plots such as those shown in Figure 3. Section 3 can be run for any combination of group/condition comparisons.

#### 7. Optical flow PCA

NOTE: Building upon the estimation of optical flow magnitude, the outputs of script2_optflow_roiselect.m (Supplementary Coding File 2) also contain information about the spatial distribution of optical flow frame-by-frame per video. In order to reduce the dimensionality of this spatial information, the provided script script4_optflow_pca.m (Supplementary Coding File 4) performs principal component analysis (PCA) on the estimated optical flow. The main inputs are the 3D optical flow matrices, previously saved as videos for each behavioral video, and the main output is one .mat file per video containing PC and explained variance information.

1. Edit Section 1 of the script to fit data structures and analysis needs.
2. Set the analysis options flags **stimnum**, **dsfactor**, and **fnfactor** as desired in Section 1. In the representative results presented, each stimulus type was analyzed separately, and the default value of **1** maintained for **dsfactor** and **fnfactor**, resulting in no downsampling in space or time.
3. Run Section 1.

#### 8. Optical flow PCA comparison

NOTE: The provided script script5_optflow_pca_analysis.m (Supplementary Coding File 5) will carry out exploratory comparisons of PCA results across groups. The main inputs are the stimulus-specific PC and explained variance information obtained from script4_optflow_pca.m (Supplementary Coding File 4), and the main outputs are statistically thresholded heat maps depicting loadings of the first PC, though other PCs can also be analyzed with this script.

**Figure 4:**
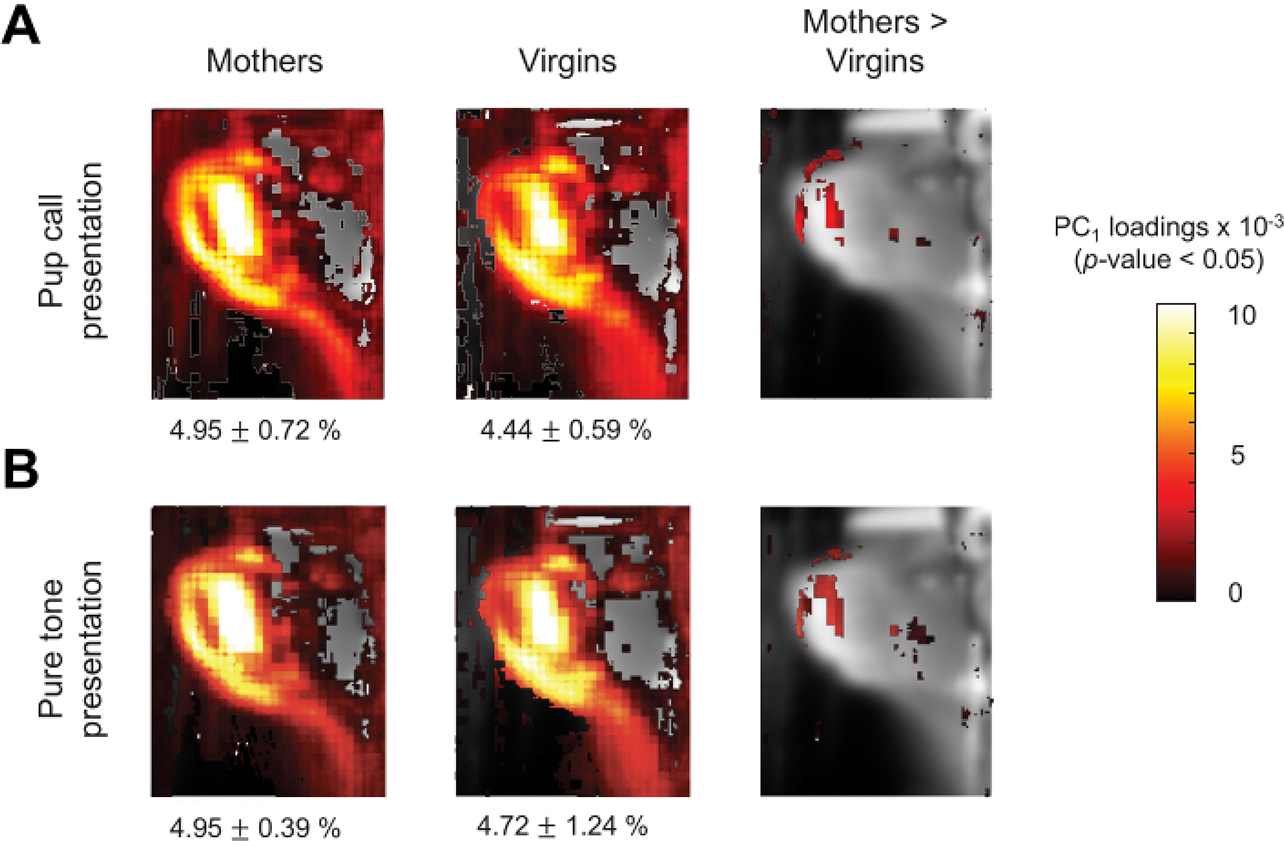
PC_1_ comparison across groups. (**A,B**) Maps depicting the loadings of the first principal component (PC_1_) of optical flow during pup calls (**A**) and pure tones (**B**) across groups, statistically thresholded at *p* < 0.05 without correction for multiple comparisons. Heat map indicates the degree to which the optical flow fluctuation of each pixel contributed to PC_1_, relative to other pixels. Small black text indicates the percent of variance explained by PC_1_ (mothers pup calls: 4.95% ± 0.72%; virgins pup calls: 4.44% ± 0.59%; mothers pure tones: 4.95% ± 0.39%; virgins pure tones: 4.72% ± 1.24% (mean ± standard deviation)).

1. Edit Section 1 of the script to fit data structures and analysis.
2. Run Section 1 and Section 2.
3. Edit Section 3 inputs, specifically the **group** and **myTitle** variables, to reflect the group analysis desired, as well as **threshT**. **threshT** represents the T-statistic threshold above which results will be considered significant, which can be calculated based on publicly-available T-statistic to *p*-value tables and the degrees of freedom.
4. Run Section 3 for each group of interest. The code should produce a group summary statistic map, such as those shown in the left and center panels of Figure 4. Adjust the range of **caxis**, and thus the color bar limits, as desired for visualization.
5. Edit Section 4 inputs, specifically the **mag** and **myTitle** variables to reflect the group comparison desired, as well as **threshT**. **threshT** represents the T-statistic threshold above which results will be considered significant, which can be calculated based on publicly-available T-statistic to *p*-value tables and the degrees of freedom.
6. Run Section 4 for each group comparison of interest. The code should produce a group comparison statistic map, as shown in the right panel of Figure 4. Adjust the range of **caxis**, and thus the color bar limits, as desired for visualization.

## Representative Results

To demonstrate the potential of this analysis pipeline, behavioral videos of head-fixed female mice — specifically mothers and virgins — were acquired while the mice were presented with auditory stimuli during functional magnetic resonance imaging (fMRI) scans. The stimuli consisted of pup calls and pure tones presented passively, thus without any instructed behavioral outputs. The pup calls were recordings of ultrasonic vocalizations emitted by 6-day-old mouse pups temporarily isolated from their nest. These pup calls typically elicit the maternal act of pup retrieval, in which the mother locates, moves toward, investigates, then picks up the pup and returns it to the safety of the nest — a behavior that virgin females typically do not exhibit^11^. Previous work has shown that pup calls elicit robust evoked activity in the primary auditory cortex of mothers but not virgins, while pure tone responses show no group differences^12^. Therefore, it was hypothesized that group differences in behavioral responses to pup calls, but not pure tones, would be identified. However, there is little literature on the specific behaviors of female mice in response to pup cues in a head-fixed environment. Therefore, no specific behavioral output was expected, making this experiment the perfect test for the proposed data-driven behavioral analyses.

Over the course of eight days, all animals were gradually habituated to experimenter handling, head fixation, and the MRI environment. Habituation to head fixation and the experimental environment is crucial for assessing behavioral responses to presented stimuli. If not properly habituated to the environment, animals may only show stress responses, washing out any stimulus-driven effects that could otherwise be parsed out *via* videography.

While the behavioral apparatus set-up was generally the same for every data acquisition session, it is possible that the camera field-of-view (FOV) shifted slightly each time an animal was head-fixed for fMRI scans (see example FOVs in Figure 1A). This was likely due to slight changes in the positioning of the camera mount, as well as individual variations in the head-post skull attachment for each animal. Therefore, the videos from the scans needed to be aligned with each other *via* linear coregistration to allow for the comparison of the spatial information they contained across the scans and animals. Coregistration was tailored to the representative data. For example, on a standard scanning day, 3–4 scans were acquired per animal, resulting in 3–4 videos per animal per day. Between each scan, there was no movement of the cradle components, including the camera and head-fixation components. Thus, once the coregistration transformation for one video per animal per day was determined, it could be applied to the other 2–3 videos of that animal on the same day. While this saved time in the coregistration step of this pipeline, spatial transformations can also be calculated for each video individually, if necessary. Three features present in each video were chosen as points for labeling and calculating spatial transformation. In the representative data shown, those three points were the center of the head-post, the frontal view of the animal’s right eye, and the profile view of the animal’s right eye (visible in a mirror placed at a 45-degree angle). Examples of video coregistration are depicted in Figure 1A,B, showing the average frame calculated from all videos taken during this experiment, both before and after coregistration, to demonstrate the effect of this step.

This pipeline relies heavily on optical flow, a computer vision method used to estimate the movement of objects in a video by estimating their apparent velocities between consecutive frames^13^. Optical flow was chosen because it allows for the quantification of movement — a proxy of behavioral responses — without any predetermined body parts or actions of interest. Additionally, this method was amenable to the limited image quality of the representative videos, which were acquired with the only MR-compatible camera accessible at the time of the experiment. In this pipeline, the Horn-Schunck algorithm of global, dense optical flow estimation is used; however, other algorithms, such as the Lucas-Kanade algorithm, can be easily employed with slight alterations to the provided MATLAB scripts^14,15,16^. Figure 2A depicts an example video frame with relative optical flow velocity vectors overlaid for each pixel. Note that the larger vectors appear in areas where one would expect movement, such as the animal’s snout and paws.

While the pipeline estimated optical flow for all videos across the full FOV, the remaining analysis steps were made much more manageable in terms of computational load and data storage needs by selecting a region of interest (ROI) within the FOV. The ROI can be selected according to the experiment and predetermined behaviors of interest or through a more data-driven approach. Given that the representative data included no specific behavioral output to track *a priori*, a data-driven approach was pursued. The standard deviation of optical flow magnitude was calculated across all videos at each pixel, as visualized in Figure 2B. Areas of highest standard deviation included contours of the animal, such as around the eye and snout, which instill confidence that the optical flow fluctuations observed were guided by animal movement and not noise in the videos. Areas of lesser standard deviation included the contours of the cradle, which could be the result of small vibrations in the camera and cradle that occurred during scanning. The MRI environment is inevitably ridden with vibrations during data acquisition due to the switching of gradients, which may manifest in optical flow fluctuations observed in reflective components of the cradle. Fortunately, these vibrations are constant throughout data acquisition, thus independent of stimulus condition and not expected to affect the results of behavioral analysis. Particularly heightened optical flow standard deviations were observed in the pixels of the mirror, corresponding to the profile view of the animal’s face, which guided the selection of the ROI for the representative data. This was also a region where behavioral responses, such as whisking and sniffing, might be expected as part of the typical behavioral repertoire of female mice seeking an isolated pup emitting pup calls^17^.

After choosing an ROI and extracting the average framewise magnitude of optical flow for all videos, the optical flow during epochs of interest could be compared across groups and conditions. Note that the first 20 seconds (s) of each video’s optical flow vector was masked for brightness stabilization; however, this can be adjusted to fit experimental needs. For each video, the framewise optical flow vector for the chosen ROI was Z-scored, then, following prior work in facial expression classification, it was lowpass filtered at 5 hertz (Hz) to account for the camera’s 30 Hz framerate being faster than any expected behavioral fluctuations^2^. Finally, the Z-scored and filtered vectors were chunked into stimulus presentation epochs to assess the effect of stimulus presentation on optical flow. For each epoch, the mean pre-stimulus baseline signal was subtracted to normalize optical flow to the pre-stimulus period. Figure 3A shows exemplary optical flow time series for two videos, one mother and one virgin, with summary group data in Figure 3B–E. Figure 3B and Figure 3D show the cumulative optical flow over time during stimulus presentation relative to baseline, while Figure 3C and Figure 3E summarize the cumulative optical flow 2.5 s after stimulus onset. Cumulative optical flow was calculated to capture overall movement over time without assuming that spontaneous behavioral responses would occur in a time-locked manner. Overall, these representative results demonstrate that pup calls, but not pure tones, evoked significantly different behavioral responses from maternal versus virgin female mice, as predicted (between-group Mann-Whitney U test: pup calls: *p* = 0.026; pure tones: *p* = 0.093). However, this stimulus effect did not survive a 2-way ANOVA, while the group effect did (stimulus: F(1,10) = 0.19, *p* = 0.67; group: F(1,10) = 8.61, *p* = 0.015). Overall, these results suggest that mothers showed higher stimulus-evoked movement compared to virgins, with the maternal response to pup calls being more consistent than to pure tones. This may reflect heightened attentiveness or stress in mothers, as well as the behavioral relevance of pup calls, which, unlike pure tones, evoke the response of pup retrieval in mothers in naturalistic settings. Taken together, these representative results suggest that optical flow estimation during external stimulus presentation can extract information regarding nuanced, spontaneous behavioral responses.

Finally, a more exploratory analysis was conducted to uncover the spatial characteristics of the behavior captured in the representative data. To investigate which pixels in the ROI fluctuated in a coordinated way during stimulus presentation, principal component analysis (PCA) was conducted on the spatial optical flow information over time. This analysis revealed pixels that contributed most to the first PC, as well as pixels that showed group differences for each stimulus condition, as depicted in Figure 4. The rightmost panels of Figure 4A,B suggest that mothers showed more movement in the nose compared to virgins during the presentation of both stimulus types. Across the two groups and two stimulus conditions, the first PC explained 5.41% ± 0.59% of the total variance in the optical flow analysis. While the mirror ROI was maintained for this part of our analysis, future analyses could expand to a broader portion of the FOV to characterize coordinated movements beyond the face in response to stimuli. For example, comparing paw movement may reveal more significant group differences, given that pup calls typically initiate pup retrieval in mothers but not virgins, and that the paws could move more freely than the head of the animal.

**Figure 5:**
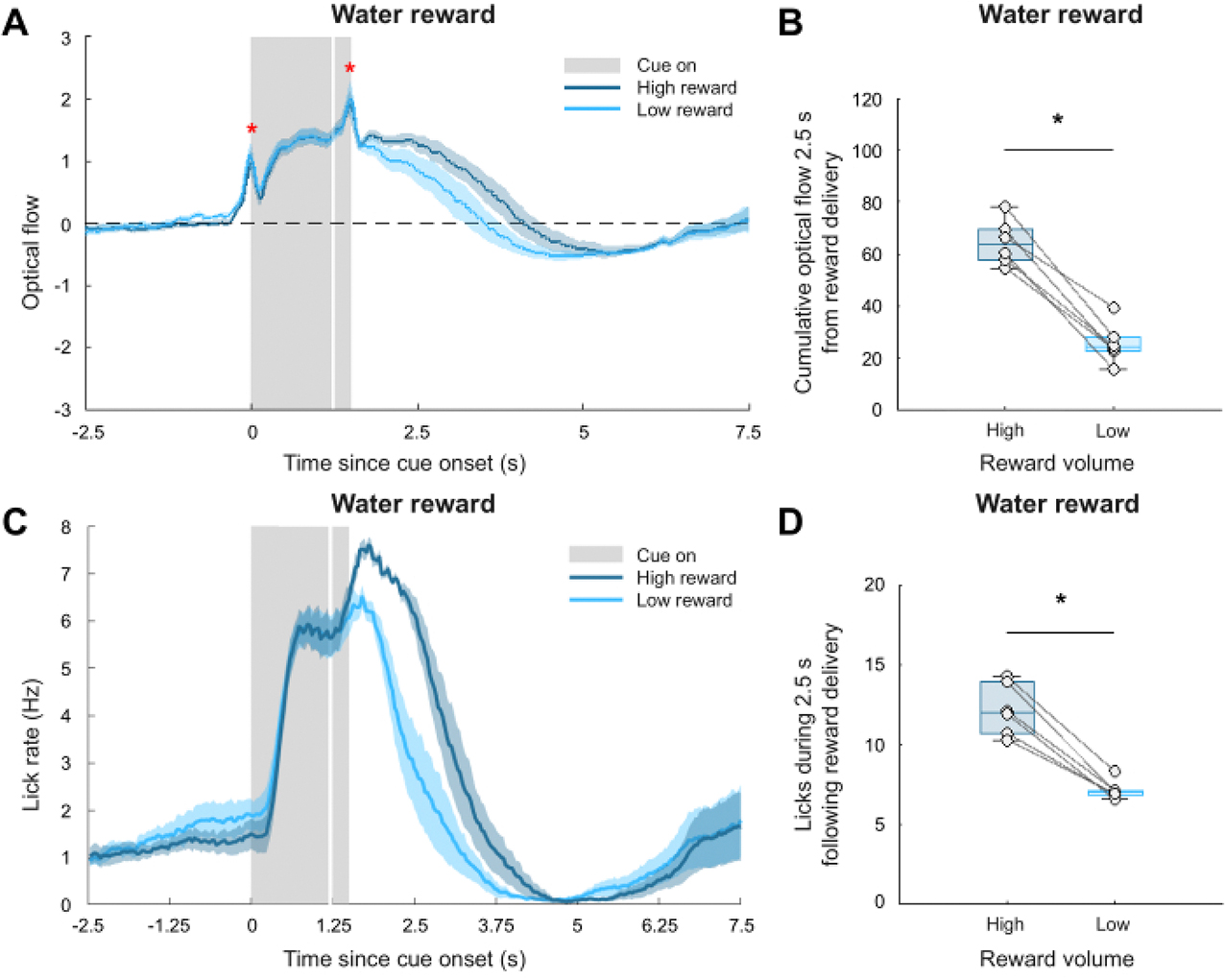
Validation of the behavioral analysis pipeline. (**A**) Average Z-scored, pre-stimulus baseline subtracted, and 5 Hz lowpass filtered time series of optical flow during high and low reward delivery. Shading represents the SEM, the white vertical line indicates reward delivery, and the red asterisks indicate temporary optical flow artifacts due to the onset and offset of the light cue. (**B**) Summary of cumulative optical flow during the first 2.5 s after reward delivery. * Indicates p < 0.05, between-condition paired Wilcoxon signed-rank test (*p* = 0.031). (**C**) Average lick rate, obtained by lickometer, during high and low reward delivery. Shading represents SEM, and the white vertical line indicates reward delivery. (**D**) Number of licks recorded during the first 2.5 s after reward delivery. * Indicates *p* < 0.05, between-condition paired Wilcoxon signed-rank test (*p* = 0.031), N = 6.

While the representative results thus far suggested that this pipeline can assess uninstructed behavioral responses to external stimuli in constrained videography environments, a question remained as to whether the observed fluctuations in optical flow truly reflected meaningful animal behavior. To answer this question, a separate validation dataset was analyzed using the same pipeline. In a separate experiment, water-restricted male mice were trained to associate a light cue with the delivery of either a 6 μL (“high reward”) or 1 μL (“low reward”) water reward. Notably, unlike the auditory stimulation experiment, this experiment had an *a priori* behavioral readout: lick rate. A lickometer was established *via* video-based detection of licks, facilitated by the analysis of pixel brightness close to the water spout. The behavioral readout supplied by the lickometer could thus be used to compare this pipeline’s behavioral readout supplied by optical flow estimation, supporting its validity in detecting spontaneous behaviors. Following video coregistration, optical flow estimation, ROI selection (once again containing the mirror FOV), and optical flow quantification, comparing optical flow magnitude revealed a significant difference between the behavioral responses to high versus low rewards. The results of this analysis are shown in Figure 5A,B, where Figure 5A shows the group averaged time series, and Figure 5B summarizes the cumulative optical flow 2.5 s after stimulus onset (between-condition paired Wilcoxon signed-rank test: *p* = 0.031). Note that optical flow values were larger compared to the spontaneous behavior recorded in the representative data, further emphasizing the challenge of evaluating uninstructed, nuanced behavioral responses. Figure 5C depicts the actual lick rate recorded by the lickometer for each of the two reward conditions, while Figure 5D shows that more licks are recorded during the high reward condition compared to the low reward condition within 2.5 s following reward delivery (between-condition paired Wilcoxon signed-rank test: *p* = 0.031). Altogether, both analyses revealed a similar trend in the comparison of high versus low reward responses, validating the presented video analysis pipeline for capturing meaningful differences in animal behavior across conditions.

Taken together, the representative results shown here suggest that the presentation of pup calls evoked significantly different responses in maternal versus virgin female mice, while pure tones did not. The analysis of a validation dataset confers confidence that the observed differences in optical flow reflect meaningful differences in behavioral responses to external stimuli.

## Discussion

The presented behavioral analysis pipeline allows for the extraction of valuable information from videos of animals exhibiting uninstructed behaviors in response to passively presented stimuli. These representative behavioral videos were acquired in conjunction with whole-brain functional magnetic resonance imaging (fMRI) data, for which multiple constraints were overcome to capture behavioral responses in the MRI environment. Employing the method of optical flow estimation, differences in how mothers versus virgins respond to pup calls—but not pure tones—were revealed, supporting the initial hypothesis^13,14,15,16^. Taking advantage of dimensionality reduction of the optical flow information, this finding was extended to an exploratory analysis of the spatial distribution of these behaviors.

This analysis pipeline is widely amenable to various experimental paradigms. It is completely agnostic to the type of neural data acquired and does not require excessive computational power or specialized equipment. The main experimental requirement is that videography data be recorded with the relevant timestamps of stimulus presentation onsets. Furthermore, by providing the MATLAB scripts used to perform the demonstrated analyses, the implementation of this pipeline should be fairly straightforward, even for those with minimal MATLAB experience. The general steps, as described in the protocol, consist of video coregistration, optical flow estimation, region-of-interest (ROI) selection, ROI-specific optical flow quantification, optical flow magnitude comparison, principal component analysis (PCA), and PCA comparison. At each step, the pipeline can be modified to fit experimental and analytical needs, as stated in the protocol and highlighted with comments throughout the code.

There are several limitations to keep in mind when employing the presented video analysis pipeline. First, the design of the behavioral apparatus should be carefully considered. If it is unclear what behavioral responses can be expected, it may benefit future analyses to have as many viewpoints of the animal as possible. Therefore, installing one or multiple mirrors to simultaneously capture the animal’s frontal and profile views is recommended, as shown in the example video frames in Figure 1A. Second, it is recommended that the illumination provided for video acquisition is bright enough to see all relevant features of the field-of-view (FOV), but not so bright as to reach saturation. In Figure 1A, it can be observed that the frontal view of the mouse nose is at a point of near saturation, making it difficult to capture any fluctuations in brightness that would indicate movement. This may have contributed to the lack of optical flow variation observed in this part of the FOV, as shown in Figure 2B. Fortunately, the profile view of the nose did not face this problem, allowing for the analysis of the mirror ROI. Additionally, the possibility of saturation should be considered if visual stimuli are involved in the experiment. In the validation dataset experiment, a light cue used to signal the delivery of a water reward introduced temporary saturation in the acquired videos, posing a temporary artifact in the optical flow estimation. This artifact can be observed in Figure 5A, where the two sharp peaks in the optical flow traces for both reward conditions align exactly with when the light cue was turned on and off. This artifact rendered these short time windows difficult to analyze with the presented pipeline, and had it not been present, the results presented in Figure 5B may have been even more significant. Constant scene illumination is a necessary assumption for most optical flow algorithms^14,16^. Therefore, this video analysis pipeline is not recommended for experiments in which the illumination of the environment changes drastically, both within and between trials. Third and finally, the sufficient habituation of animals to the recording environment is a key component of any experiment that will take advantage of this pipeline. If not properly habituated, animals may show significant stress responses, washing out any subtle stimulus-driven effects that could otherwise be parsed out *via* video analysis.

Moving forward, this behavioral analysis pipeline can be employed to facilitate the coordinated evaluation of simultaneously recorded behavioral and neural responses. As shown, quantitatively assessing uninstructed behaviors allows for the evaluation of the salience of stimuli in a complex recording environment, as well as testing for different behavioral responses across experimental groups and conditions. Further analyses can be conducted to explore individual differences or trial-by-trial trends in behavioral responses such that the corresponding neural activity can be examined. Additionally, incorporating PCA to obtain spatial information about stimulus-evoked movement can be used to further investigate whether specific types of behavioral sequences observed during stimulus presentation differ across groups and conditions. This analysis pipeline can be easily extended to various neural recording methods, providing versatile behavioral monitoring in constrained environments. By investigating how external stimuli are represented in both the brain and behavior, researchers are primed to obtain a more comprehensive understanding of how the perception of stimuli affects internal states and their external manifestations.

## Supplementary Material

Video 1 **Video 1: Example video 1.** PCR_Br011_20231015_1842_output.avi.

Video 2 **Video 2: Example video 2.** PCR_Br014_20231015_1722_output.avi.

Video timestamps 1 **Supplementary File 1: Event timing file for Video 1.** PCR_Br011_20231015_1842_output_videoTimestamps.mat.

Video timestamps 2 **Supplementary File 2: Event timing file for Video 2.** PCR_Br014_20231015_1722_output_videoTimestamps.mat.

Script 1 **Supplementary Coding File 1: script1_videocoreg.m.** This script spatially aligns all videos to each other via coregistration.

Script 2 **Supplementary Coding File 2: script2_optflow_roiselect.m.** This script estimates optical flow for the full FOV of each transformed video and allows for the selection of an ROI for the rest of the pipeline.

Script 3 **Supplementary Coding File 3: script3_optflow_analysis.m.** This script compares optical flow magnitude across different groups/conditions for the chosen ROI.

Script 4 **Supplementary Coding File 4: script4_optflow_pca.m.** This script performs PCA on the estimated optical flow of the chosen ROI.

Script 5 **Supplementary Coding File 5: script5_optflow_pca_analysis.m.** This script compares PCA results across groups/conditions for the chosen ROI.

## Figures and Tables

**Materials:** 

Name	Company	Catalog Number	Comments
MATLAB Computer Vision Toolbox	MathWorks		https://www.mathworks.com/products/computer-vision.html
MATLAB Image Processing Toolbox	MathWorks		https://www.mathworks.com/products/image-processing.html
MATLAB software	MathWorks		https://www.mathworks.com/products/matlab.html
MR-compatible camera “12M-i” with integrated LED light	MRC Systems GmbH	12M-i	https://www.mrc-systems.de/downloads/en/mri-compatible-cameras/manual_mrcam_12m-i.pdf
